# Emergence and circulation of azole-resistant *C. albicans*, *C. auris* and *C. parapsilosis* bloodstream isolates carrying Y132F, K143R or T220L Erg11p substitutions in Colombia

**DOI:** 10.3389/fcimb.2023.1136217

**Published:** 2023-03-21

**Authors:** Andres Ceballos-Garzon, Ana Peñuela, Sandra Valderrama-Beltrán, Yerly Vargas-Casanova, Beatriz Ariza, Claudia M. Parra-Giraldo

**Affiliations:** ^1^ Unidad de Proteomica y Micosis Humanas, Grupo de Investigación en Enfermedades Infecciosas, Departamento de Microbiología, Pontificia Universidad Javeriana, Bogotá, Colombia; ^2^ Laboratorio Clínico, Área de Microbiología, Hospital Universitario San Ignacio, Bogotá, Colombia; ^3^ Unidad de Infectología, Departamento de Medicina Interna, Facultad de Medicina, Hospital Universitario San Ignacio, Pontificia Universidad Javeriana, Bogotá, Colombia

**Keywords:** Candida species, bloodstream infections, fluconazole resistance, ERG11, Y132F, Colombia

## Abstract

**Methods:**

Over a four-year period, 123 Candida bloodstream isolates were collected at a quaternary care hospital. The isolates were identified by MALDI-TOF MS and their fluconazole (FLC) susceptibility patterns were assessed according to CLSI guidelines. Subsequently, sequencing of ERG11, TAC1 or MRR1, and efflux pump activity were performed for resistant isolates.

**Results:**

Out of 123 clinical strains,C. albicans accounted for 37.4%, followed by C. tropicalis 26.8%, C. parapsilosis 19.5%, C. auris 8.1%, C. glabrata 4.1%, C. krusei 2.4% and C. lusitaniae 1.6%. Resistance to FLC reached 18%; in addition, a high proportion of isolates were cross-resistant to voriconazole. Erg11 amino acid substitutions associated with FLC-resistance (Y132F, K143R, or T220L) were found in 11/19 (58%) of FLCresistant isolates. Furthermore, novel mutations were found in all genes evaluated. Regarding efflux pumps, 8/19 (42%) of FLC-resistant Candida spp strains showed significant efflux activity. Finally, 6/19 (31%) of FLC-resistant isolates neither harbored resistance-associated mutations nor showed efflux pump activity. Among FLC-resistant species, C. auris 7/10 (70%) and C. parapsilosis 6/24 (25%) displayed the highest percentages of resistance (C. albicans 6/46, 13%).

**Discussion:**

Overall, 68% of FLC-resistant isolates exhibited a mechanism that could explain their phenotype (e.g. mutations, efflux pump activity, or both). We provide evidence that isolates from patients admitted to a Colombian hospital harbor amino acid substitutions related to resistance to one of the most commonly used molecules in the hospital setting, with Y132F being the most frequently detected.

## 1 Introduction

Invasive fungal infections (IFIs) due to *Candida* species are a frequent and life-threatening condition in hospital settings worldwide, and are often associated with high morbidity and mortality (Koehler et al., 2019). *Candida albicans* is the most isolated species, with close to a 30% mortality rate in candidemia (Pappas et al., 2018). However, non-*albicans Candida* species (NACS) such as *C. glabrata* and *C. parapsilosis* have emerged as a common cause, becoming the second or third most frequent species depending on geography, patient underlying condition, and age. For these species, the associated mortality rate is about 50% and 28%, respectively (Pemán et al., 2005; Lamoth et al., 2018). Another important NACS causing candidemia is *C. auris*, whose mortality rate ranges from 40% to 60% according to some studies, despite the fact its prevalence is unclear (Chen et al., 2020).

While current therapeutic options for IFIs are limited to only three classes of drugs (i.e., polyenes, azoles and echinocandins), the emergence of resistant strains to some of these molecules is even more concerning. For decades, azoles have been the most frequently used antifungal for treating *Candida* infections (Sheehan et al., 1999; Carradori et al., 2022). Although treatment with azoles can be effective, long-term use of fluconazole (FLC) has led to the emergence of *Candida* strains with decreased susceptibility. In the case of *Candida* spp, the molecular mechanisms behind FLC-resistance have been relatively well characterized (Morio et al., 2017; Carolus et al., 2021).

Unlike *C. auris* which is often resistant to FLC, *C. albicans* and *C. parapsilosis* isolates were thought to be universally susceptible to FLC, but recent studies show an increased resistance. For example, a multicenter laboratory-based survey of candidemia conducted in South Africa indicates that more than half of *C. parapsilosis* isolates 62% (332/531) are resistant to FLC (Govender et al., 2016). In addition, studies in Brazil, India, Kuwait, South Korea, Spain, Turkey, and the United States, as well as a recent global study, confirmed the emergence of FLC-resistance in *C. parapsilosis* (Berkow et al., 2015; Asadzadeh et al., 2017; Arastehfar et al., 2020; Castanheira et al., 2020; Díaz-García et al., 2022). Regarding *C. albicans*, this species exhibits lower levels of azole resistance. However, resistant isolates have been reported from many countries around the world, including Colombia (Costa-de-Oliveira and Rodrigues, 2020; Rojas et al., 2020; Ceballos-Garzon et al., 2020b).

The main FLC-resistance mechanisms are associated with *i*) up-regulation of drug transporters, *ii*) alteration or up-regulation of the gene encoding the enzyme being targeted, which decreases binding affinity for the drug and increases concentration of the enzyme target, *iii*) alterations in the ergosterol synthetic pathway and *iv*) activation of pathways involved in the stress response, such as the Ras/cAMP/PKA pathway, calmodulin/calcineurin pathway (CaM/CaL), and mitogen-activated protein kinase (MAPK) signaling pathways (Shapiro et al., 2011; Pristov and Ghannoum, 2019). However, the most studied of them are described below.

The ATP-binding cassette (ABC) and the major facilitator superfamily (MFS) transporters are responsible for lowering the accumulation of azoles inside the yeast cell by translocating compounds actively across the cell membrane (Costa-de-Oliveira and Rodrigues, 2020). Overexpression of genes encoding drug transporters, e.g., Cdr1/2-ABC and Mdr1/Flu1-MFS, among resistant isolates of *Candida* species is predominantly due to gain-of-function (GOF) mutations in genes encoding zinc cluster transcription factors, such as *TAC*1 (transcriptional activator of CDR genes) and *MRR*1 (multidrug resistance regulator). For instance, GOFs in *TAC*1 (T225A, R693K, A736V, H741, N972D, G980E, N997D) and *MRR*1 (I283R, R479K, G583R, V854A, K873N), lead to overexpression of *CDR*1 and *MDR*1 in *C. albicans and C. parapsilosis*, respectively (Coste et al., 2006; Liu et al., 2020).

Overexpression of *ERG*11, the gene encoding lanosterol 14α-demethylase, the azole target, contributes directly to resistance as the increased abundance of the target requires higher drug doses for inhibition. Activating mutations in the gene encoding the transcription factor Upc2, which up-regulates most ergosterol biosynthesis genes, and the formation of an isochromosome with two copies of the left arm of chromosome 5 [i(5L)], or by duplication of the whole chromosome, on which *ERG*11 resides, are responsible for *ERG*11 overexpression (Cowen et al., 2014). Furthermore, point mutations in the *ERG*11 alter the 3D conformation of Erg11 and reduce its affinity for FLC. Some of the most frequent amino acid substitutions reported are Y132F and K143R substitutions, described in *C. albicans, C. parapsilosis* and *C. auris* (Berkow et al., 2015; Flowers et al., 2015; Chow et al., 2020).

Although *Candida* isolates from individual institutions may not be representative of the data of a country, such studies can provide a useful baseline snapshot of species distribution and antifungal susceptibility for candidemia in resource-limited settings (Govender et al., 2016). In Colombia, there is a lack of data about antifungal resistance and its molecular mechanisms in *Candida* spp. Therefore, this study aimed to investigate the prevalence of resistance and to describe the mechanisms behind FLC-resistance in a collection of bloodstream isolates.

## 2 Materials and methods

### 2.1 Ethics approval and consent to participate

The research and ethics committee of the Hospital Universitario San Ignacio (HUSI) approved this study (no. FM-CIE-8053-14). All patients are anonymized and only the code of isolates was transferred for this investigation. Therefore, no informed consent was required.

### 2.2 Study design

The study was a single-center retrospective analysis. One hundred twenty-three bloodstream isolates of *Candida* spp. obtained from 123 hospitalized patients (2016-2020) of the San Ignacio Hospital in Bogota, Colombia were included. Prior to storage at −80°C, yeast from blood cultures submitted for routine work-up to the Clinical Microbiology Laboratory were primarily identified using MicroScan (MicroScan WalkAway-96 Plus, Siemens, Deerfield, IL, USA) or VITEK 2 system (bioMérieux, Marcy-l’Etoile, France), and further characterized (this study) using the MALDI-TOF Biotyper system (Bruker Daltonik, Bremen, Germany).

### 2.3 MALDI-TOF MS

Isolates were streaked from a glycerol stock onto Sabouraud dextrose agar (SDA) and grown for 24–36 h at 35°C. Protein extraction was performed using formic acid/ethanol method, according to the Bruker Daltonics’ protocol. The protein mass spectra were analysed using the Flex Control software and the MALDI Biotyper version 3.1 7311 reference spectra (main spectra) (Bruker Daltonics, Bremen, Germany). MALDI-TOF MS results were obtained according to the manufacturer’s technical specifications, as follows: correct genus and species identification (≥2.0), correct genus identification (1.7–2.0), and no reliable identification (< 1.7). All clinical isolates had a score above 2.0 (Ceballos-Garzon et al., 2020a).

### 2.4 Antifungal susceptibility testing

Susceptibility to FLC (Sigma-Aldrich, St. Louis, MO, USA) was conducted using the Clinical and Laboratory Standards Institute broth microdilution method (CLSI-BMD), following the M27-A3 document (CLSI, 2008). Quality control was ensured by testing the CLSI-recommended strains *C. parapsilosis* ATCC 22019 and *C. krusei* ATCC 6258. For NACS isolates the CLSI breakpoints were applied (resistance to FLC was set at *C. albicans, C. tropicalis, and C. parapsilosis ≥*8µg/mL and *C. glabrata ≥*64µg/mL) (CLSI, 2018). In the case of *C. auris*, the FLC breakpoint recommended by the US Centers for Disease Control and Prevention (CDC) was used (≥32 µg/mL) (CDC, 2020). The MIC data obtained under routine conditions for amphotericin B (AMB), caspofungin (CAS), itraconazole (ITC), and voriconazole (VRC) by Etest^®^ (bioMérieux, Marcy-l’Étoile, France) and VITEK^®^2 (bioMérieux) are presented in **Table S1**.

### 2.5 Sequencing analysis of Erg11, Tac1 and Mrr1-encoding genes

All isolates displaying resistance to FLC, and one susceptible isolate of each species were subjected to a single-tube PCR method to amplify and sequence the coding region of the *ERG11*, *TAC*1 or *MRR*1 genes (both strands) using the primers indicated in **Table S2**. The PCR products were purified and sequenced using a SeqStudio genetic analyzer capillary sequencer (Applied Biosystems). The sequencing results were analyzed by BLAST and compared with the published GenBank sequences: *C. albicans* AY856352.1 (*ERG*11), DQ393587 (*TAC*1), and *C. parapsilosis* GQ302972 (*ERG*11), HE605205 (*MRR*1). For *C. auris*, sequences download from the Candida genome database (candidagenome.org) were used, i.e., B9J08_001448 (*C. auris* B8441, *ERG*11) and B9J08_004820 (*TAC*1b). All sequences (FLC-susceptible and resistant clinical isolates plus reference strains) were aligned, and the dataset was used to construct a Neighbor-Joining phylogenetic tree using Maximum Composite Likelihood settings by using Molecular Evolutionary Genetics Analysis Version 11 (MEGA11) (Saitou and Nei, 1987; Tamura et al., 2021). Codon positions included were 1st + 2nd + 3rd + Noncoding. All positions containing gaps and missing data were eliminated. Evaluation of branch support was performed by Bootstrap statistical analysis with 1000 replicates (Tamura et al., 2021).

### 2.6 Analysis of rhodamine 6G efflux

The accumulation of R6G in growing *Candida* cells correlates inversely with the mRNA expression level of the ABC transporter Candida drug resistance 1 (CDR1), therefore the levels of intracellular accumulation of R6G can be used for the identification of azole-resistant strains. ABC transporter-mediated efflux was determined using rhodamine 6G (Sigma-Aldrich, USA) as previously described by Gbelska and coworkers in a step-by-step protocol (Gbelska et al., 2017). The fluorescence of the released R6G was measured at 530 nm, with an emission at 560 nm in an automated plate reader (Model 550 Microplate Reader Bio-Rad, Milan, Italy). Measurements were made before (basal) and after the addition of 20 mM glucose. Using a R6G calibration curve, the fluorescence intensity was converted into concentration.

### 2.7 Statistics

Antifungal susceptibility and 6G efflux were performed in triplicate, in three independent experimental sets. The results of the efflux pump activity were analyzed statistically by the Analysis of Variance One-Way ANOVA using GraphPad Prism version 9 software. In all analyses, p values of 0.05 or less were considered statistically significant.

## 3 Results

### 3.1 Identification and Antifungal susceptibility testing of clinical isolates

**Figure 1:**
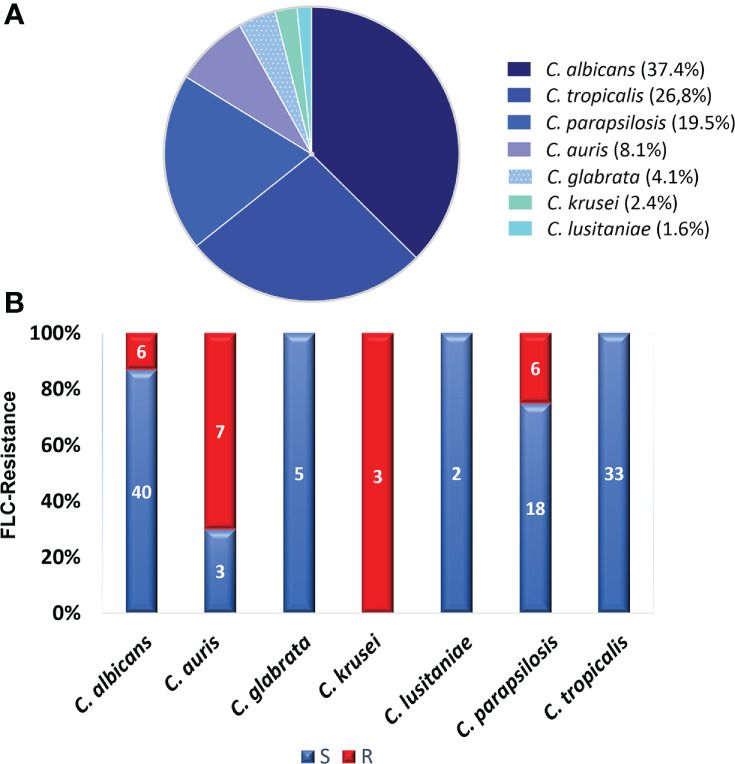
Species distribution and overall fluconazole susceptibility results. **(A)** Distribution of the 123 species identified. **(B)** Percentage of fluconazole resistance. The number of isolates is indicated inside the bars. Susceptible (S) isolates are depicted in blue and resistant (R) isolates in red.

The *Candida* species distribution from the 123 blood samples was as follows: *C. albicans*, 46 (37.4%); *C. tropicalis*, 33 (26.8%); *C. parapsilosis*, 24 (19.5%); *C. auris*, 10 (8.1%); *C. glabrata*, 5 (4.1%); *C. krusei*, 3 (2.4%); and *C. lusitaniae*, 2 (1.6%). Although *C. albicans* was the most prevalent species, accounting for 37.4%, the NACS group comprised 62.6% of the isolates identified. Concerning susceptibility, **Table 1** shows the geometric MICs, the MIC ranges, the MIC50, and MIC90 distributions of FLC against 123 *Candida* spp strains. The MIC_90_ values (MICs at which ≥90% of strains are inhibited) for the four most frequent species found were: *C. albicans* 128µg/mL, *C. tropicalis* 2µg/mL, *C. parapsilosis* 32µg/mL and *C. auris >*128µg/mL. As expected, *C. auris* presented the highest MIC_90_ values. In addition, the range of FLC MICs was narrower for *C. auris* and *C. glabrata* than for the other species. Both, *C. auris* and *C. glabrata* had the highest geometric mean values: 39 and 42 respectively. On the other hand, *C. tropicalis* showed reduced susceptibility to FLC (MIC_90 =_ 2µg/mL). When the CLSI and CDC breakpoints were applied, 22 out of 123 (18%) isolates displayed *in vitro* resistance to FLC, among them, six of *C. albicans*, six of *C. parapsilosis* and seven of *C. auris* (19/22). Moreover, three isolates of *C. krusei -*as *C. krusei* is assumed to be intrinsically resistant to FLC-, were not included within the molecular study. In contrast, all isolates of *C. tropicalis*, *C. glabrata*, and *C. lusitaniae* were FLC-susceptible **Figure 1**. Regarding VRC MICs (data obtained by Etest), 10 out of 19 FLC-resistant isolates, excluding *C. krusei*, were VRC cross-resistant. The highest MICs were observed for *C albicans* (32 µg/mL) **Table S3**. Considering the MIC data obtained under routine conditions for AMB (mean: 0.3 µg/mL) and CAS (mean 0.06 µg/mL) all strains showed low MICs values (susceptible) **Table S1**.

**Table 1:** Antifungal activity of fluconazole drug against *Candida* spp (n = 123) performed by CLSI.

Species	Range	GM	Number (and cumulative percentage) of Candida spp strains with MIC µg/mL																	Total
			≤0.015	0.03	0.06	0.125	0.25	0.50	1		2		4	8	16	32	64	128	>128	
*C. albicans*	≤0.015 - >128	0.61	4(9)	1(11)		1(13)	**19(54)**	9(74)	3(80)		1(83)		2(87)					4(96)	2(100)	46
*C. auris*	2 - >128	42.2									1(10)			2(30)			**2(50)**		5(100)	10
*C. glabrata*	1 - 8	39.3							1(20)		1(40)		**2(80)**	1(100)						5
*C. krusei*	≤0.015 - 4	12.6												1(33)		**2(100)**				3
*C. lusitaniae*	2	2									**2(100)**									2
*C. parapsilosis*	≤0.015 - 64	1.9	2(8)			1(13)		3(25)	5(46)		**3(58)**		4(75)		1(79)	3(92)	2(100)			24
*C. tropicalis*	0.06 - 4	0.5			4(12)		6(30)	**10(61)**	3(70)		8(94)		2(100)							33
**Overall**																				**123**

GM, Geometric mean; MIC_50_ and MIC_90_ values (MICs at which ≥50% and ≥90% of the strains are inhibited, respectively) are depicted in bold and underlined, respectively.

### 3.2 Detection of mutations in Erg11, Tac1 and Mrr1-encoding genes of 19 FLC-resistant isolates

**Figure 2:**
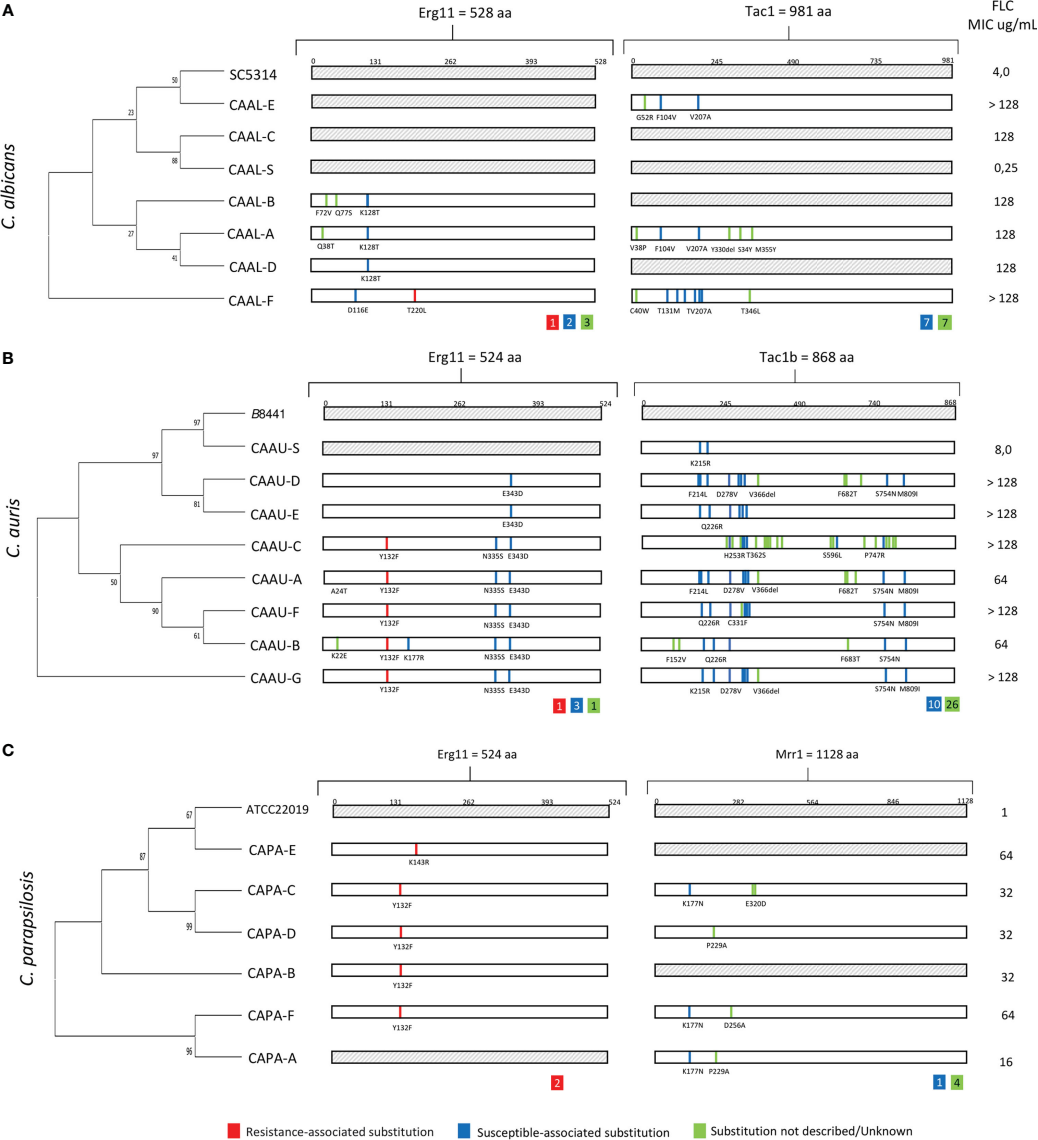
Phylogenetic tree and amino acid substitutions found in fluconazole-resistant isolates of **(A)** *C albicans* (CAAL), **(B)** *C auris* (CAAU) and **(C)** *C parapsilosis* (CAPA). aa., amino acids; FLC, fluconazole; MIC, minimal inhibitory concentration; CA-S., *Candida* FLC-susceptible. Inside the colored squares, the number of substitutions found is indicated.

By comparing the *ERG*11 coding region of *C. albicans* (CAAL, A-F)*, C. auris* (CAAU, A-G) *and C. parapsilosis* (CAPA, A-F) FLC-resistant isolates with that of our FLC-susceptible and the published wild-type sequences, we identified 20, 16, and three mutations, respectively. As expected, some silent mutations that do not change the protein sequence were identified (data not shown). The remaining *ERG*11 mutations which resulted in amino acid changes are shown in, **Table 2**, **Figure 2**. Among the nonsense mutations (*C. albicans* 6; *C. auris* 5; and *C. parapsilosis* 2), three amino acid substitutions related with FLC-resistance (T220L, Y132F, K143R) were found from which Y132F was the most detected. Additionally, five amino acid substitutions previously described in FLC-susceptible isolates were observed (D116E, K128T, K177R, N335S, E343D). To the best of our knowledge, four amino acid substitutions (K22E, Q38T, F72V, Q77S) have not been previously reported. Overall, eight of the 19 FLC-resistant isolates did not have resistance-associated substitutions.

**Table 2:** Amino acid substitutions found in 19 FLC-resistant isolates.

Isolate	CMI (ug/mL)	Erg11 amino acid substitutions	Tac1 amino acid substitutions
CAAL-A	128	Q38T, K128T	V38P, F104V, V207A, Y330del, S345Y, M355Y, M36I
CAAL-B	128	F72V, Q77S, K128T	–
CAAL-C	128	–	–
CAAL-D	128	K128T	–
CAAL-E	>128	–	G52R, F104V, V207A
CAAL-F	>128	D116E, **T220L**	C40W, T131M, M170V, F189S, S199N, R206H, V207A, T346L
Isolate	CMI (ug/mL)	Erg11 amino acid substitutions	Tac1b amino acid substitutions
CAAU-A	64	**Y132F**, N335S, E343D	F214L, K215R, Q226R, D278V, C334F, L335S, S339A, V366del, F682T, F683L, T695S, S754N, M809I
CAAU-B	64	K22E, **Y132F**, K177R, N335S, E343D	F152V, D167A, K215R, Q226R, D278V, F683T, S754N, M809I
CAAU-C	>128	**Y132F**,N335S, E343D	H253R, S267W, V269R, L270V, D278V, E305K, C331F, C334F, L335S, S339A, T362S, R402W, Y403D, A404T, D422R, C435R, S596L, Y608S, P747R, S754N, P756R, S757A, M766S, H767G
CAAU-D	>128	E343D	F214L, K215R, Q226R, D278V, C334F, L335S, S339A, V366del, F682T, F683L, T695S, S754N, M809I
CAAU-E	>128	E343D	K215R, Q226R, D278V, C331S, C334F, L335S, S339A
CAAU-F	>128	**Y132F**, N335S, E343D	K215R, Q226R, D278V, C331F, C334F, L335S, S339A, S754N, M809I
CAAU-G	>128	**Y132F**, N335S, E343D	K215R, Q226R, D278V, C334F, L335S, S339A, V366del, S754N, M809I
Isolate	CMI (ug/mL)	Erg11 amino acid substitutions	Mrr1 amino acid substitutions
CAPA-A	16	–	K177N, P229A
CAPA-B	32	Y132F	–
CAPA-C	32	Y132F	K177N, E320D, S322A
CAPA-D	32	Y132F	P229A
CAPA-E	64	K143R	–
CAPA-F	64	Y132F	K177N, D256A

Amino acid substitutions: in red, substitution associated with resistance; blue, substitution associated with susceptibility; black, substitution not described/unknown.

On the other hand, except for isolate CAAL-A, which harbored a K128T substitution in only one *ERG*11 allele, other isolates were homozygous for mutations in the *ERG*11 allele. In the phylogenetic relationship among FLC-resistant, susceptible isolates and reference strains, a cluster of isolates carrying the substitutions Y132F in *C. auris* was observed, as well as in the FLC-resistant *C. albicans* isolates harboring K128T substitution. In addition, susceptible and resistant isolates without resistance-associated *ERG*11 mutations (i.e., T220L, Y132F) from these species were clustered **Figures 2A, B**.

Concerning *TAC1* (*C. albicans* and *C. auris*) and *MRR1* (*C. parapsilosis*) genes, no mutations previously associated with FLC-resistance were found. However, 17 Tac1 (*C. albicans*, n=7; *C. auris*, n=10) and Mrr1 (*C. parapsilosis*, n*=*1) amino acid substitutions previously described in FLC-susceptible isolates were found. Additionally, there were 37 unreported substitutions in Tac1 (*C. albicans*, n=7; *C. auris, n=*26) and Mrr1 (*C. parapsilosis, n=4*) **Table 2**.

### 3.3 Efflux pumps activity

**Figure 3:**
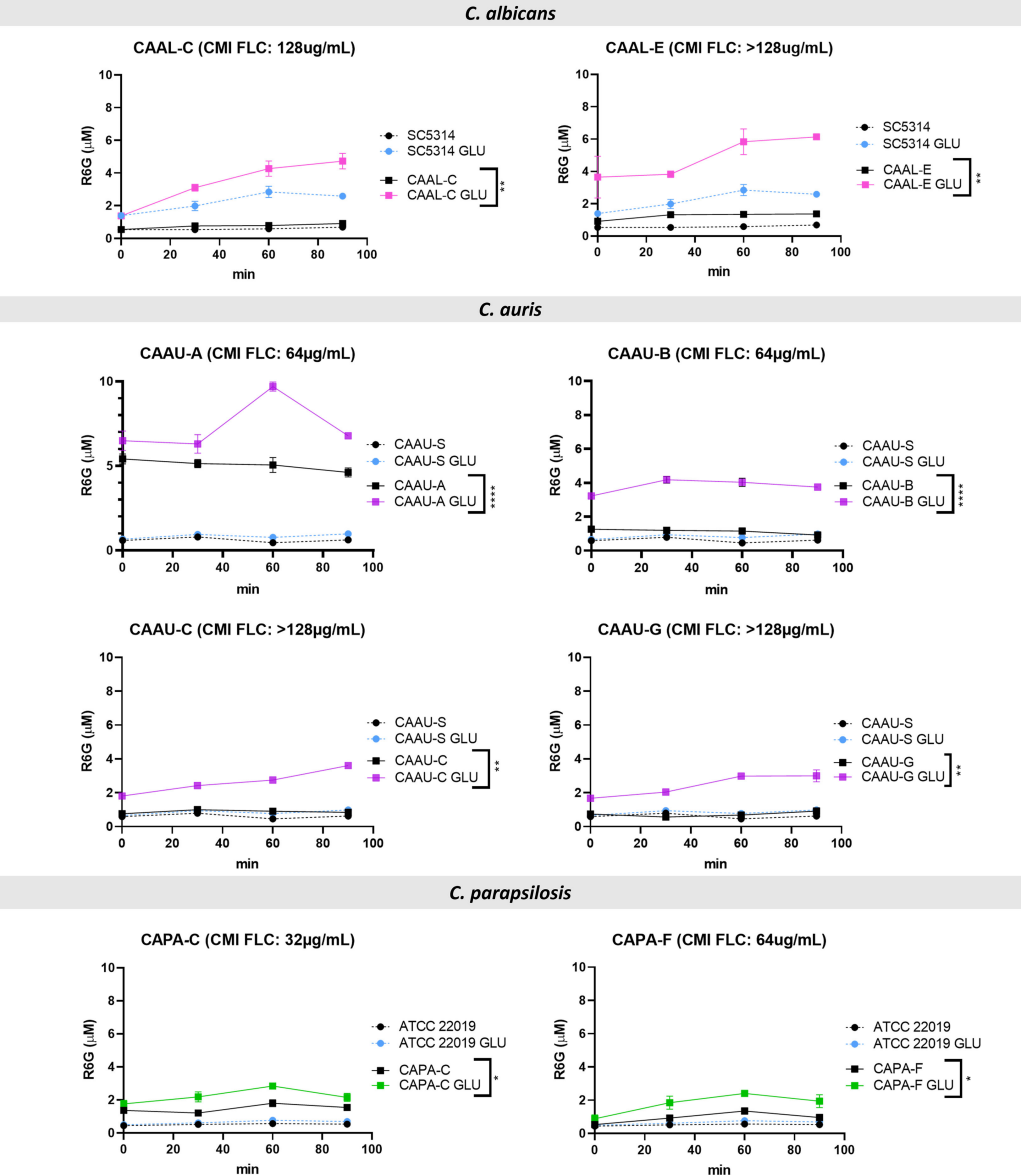
Rhodamine 6G (R6G) efflux over time among fluconazole-resistant isolates. Colored lines indicate the concentration of R6G released after the addition of 20 mM glucose (GLU). Data are means ± SD from three experiments. *p < 0.05, **p < 0.01, ***p < 0.001. ****p≤ 0.0001. FLC, fluconazole; MIC, minimal inhibitory concentration.

To gain further insights into the mechanisms of azole resistance in the clinical isolates the activity of efflux pumps was evaluated using rhodamine 6G, which uses the same membrane ABC transporters (Cdr1p and Cdr2p) as FLC in *Candida*. Among the 19 FLC-resistant isolates, eight of them showed significant active efflux of rhodamine-6G after addition of glucose: two of them belonging to *C. albicans* (CAAL-C and CAAL-E); four to *C. auris* (CAAU-A, CAAU-B, CAAU-C and CAAU-G) and two to *C. parapsilosis* (CAPA-C, CAPA-F) **Figure 3**, **Figure 1S**.

Interestingly, no amino acid substitutions associated with FLC-resistance were identified in both *C. albicans* isolates (CAAL-C, 128µg/mL), (CAAL-D, 128µg/mL). Regarding the remaining isolates that showed efflux pump activity, *C. auris* (CAAU-A, CAAU-B, CAAU-C, CAAU-G) and *C. parapsilosis* (CAPA-C, CAPA-F) harbored the Y132F substitution.

**Figure 4:**
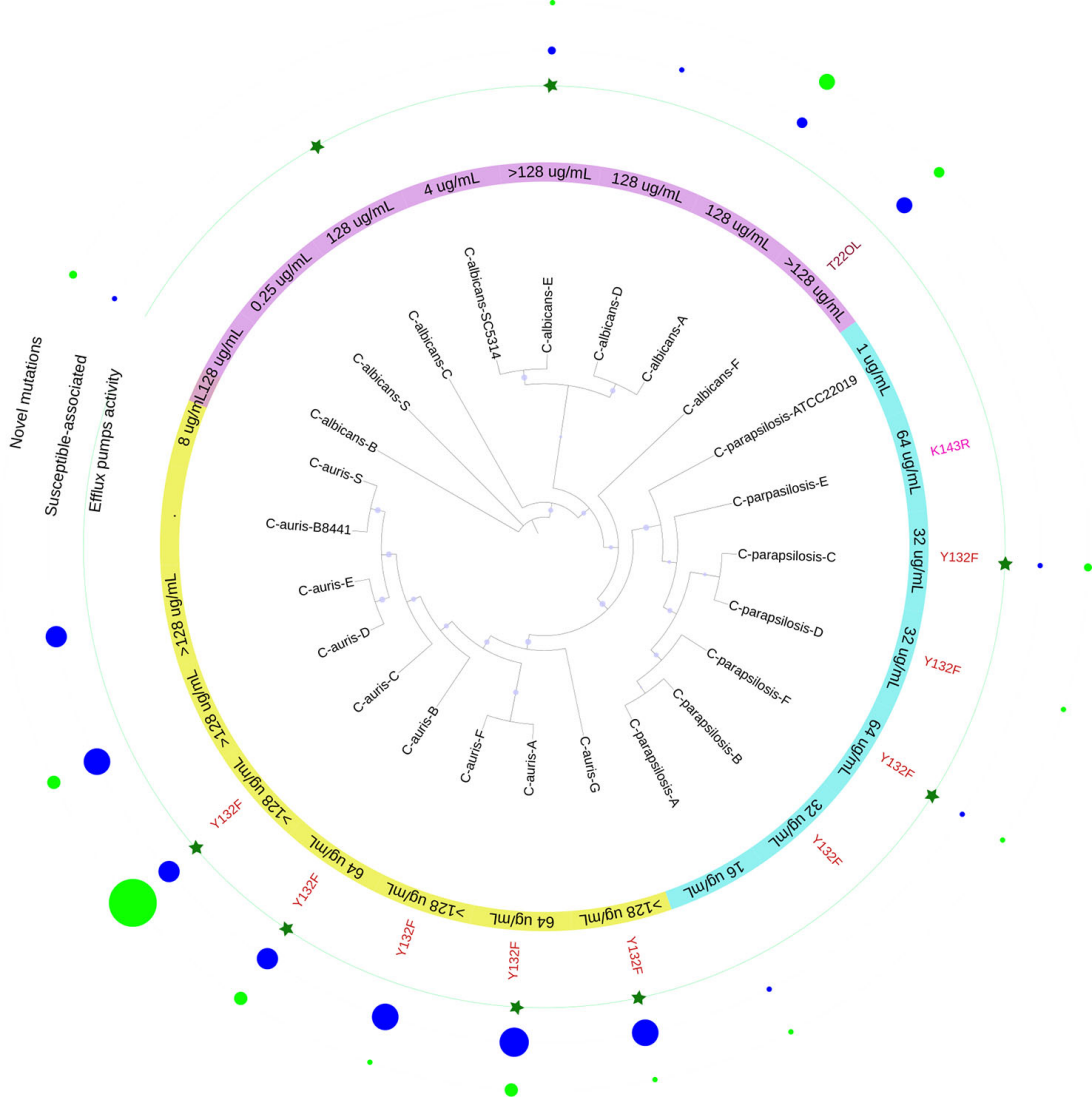
Schematic representation of the results obtained in this study for the 19 FLC-resistant isolates. In purple *C. albicans*, light blue *C. parapsilosis* and yellow *C. auris* isolates with their respective MICs against FLC. In red font, the mutations found. The green stars indicate isolates with significant efflux pump activity. The size of the circle reflects the number of mutations, those not associated with FLC resistance (blue) and the new mutations (light green).

Overall, 11/19 resistant isolates harbored an amino acid substitution associated with FLC-resistance; 8/19 displayed efflux pumps activity; 6/19 had both amino acid substitutions and efflux pump activity; and 6/19 isolates did not exhibit any of the mechanisms that could explain their resistant phenotype **Figure 4**.

## 4 Discussion

**Figure 5:**
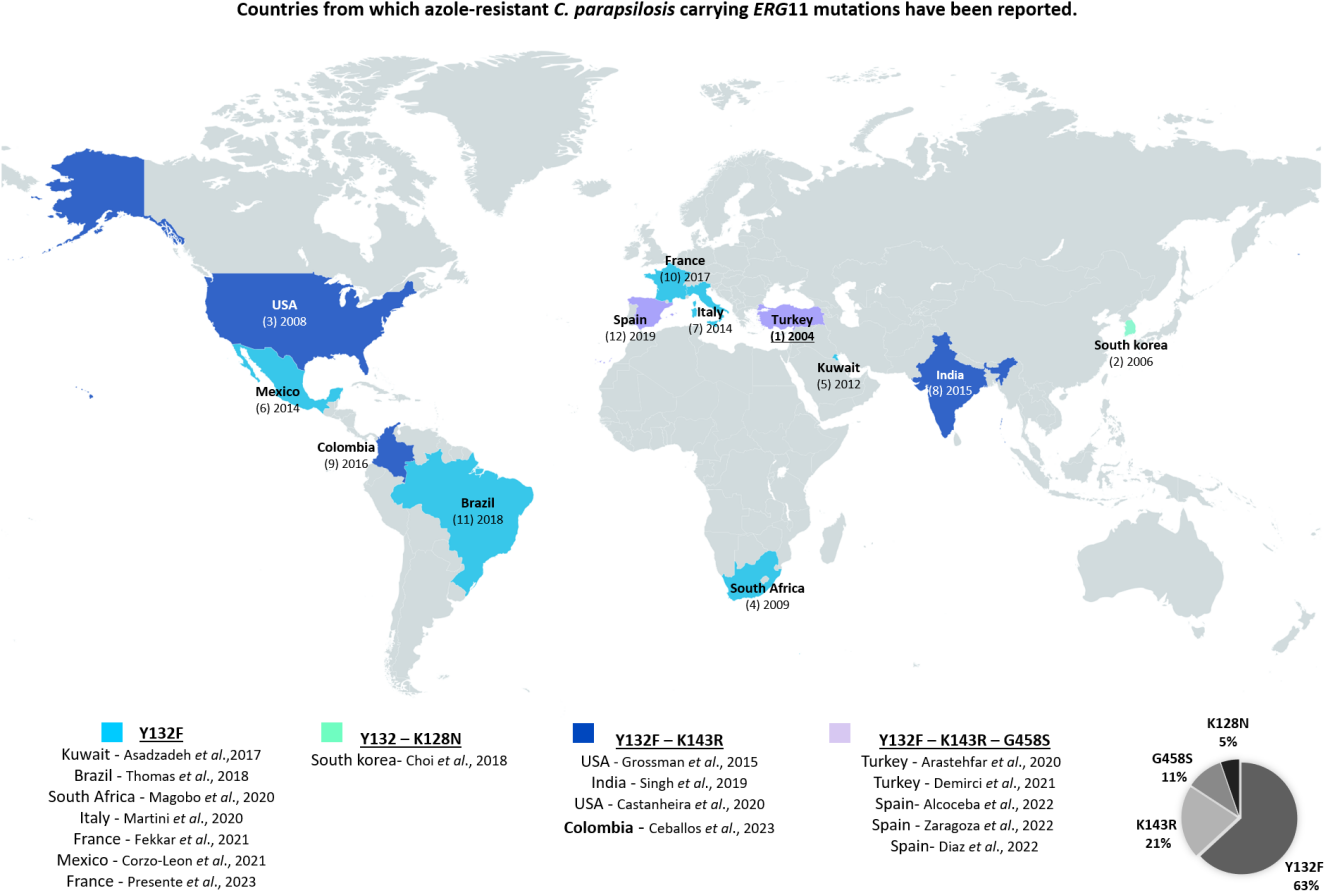
Countries from which azole-resistant *C. parapsilosis* carrying *ERG11* mutations have been reported, as of February, 2023.

In the region, a study that evaluated susceptibility of *Candida* species identified in Colombia, Ecuador, and Venezuela, found a percentage of resistance to FLC of 6.8% (De bedout et al., 2003). This shows that there is an important change in the rate of resistance, which in our study reached 18%. Nevertheless, the majority of *C. albicans* isolates were azole susceptible, thus the observed resistance percentage is mainly attributed to the presence of *C. auris*, which agrees with previous studies (Pfaller et al., 2019; Sathi et al., 2022). Furthermore, it is noteworthy that 25% of the *C. parapsilosis* isolates were resistant to FLC, which confirms the increase of resistance in this species (Escribano and Guinea, 2022) (see **Figure 5**). High azole resistance rates have been reported for this species in other single-center studies conducted in Brazil (67.9%), Italy (33%), France (9.2%), Mexico (54%), Saudi Arabia (33%), Spain (13.6%), South Africa (78%) or Turkey (26.4%) (Arastehfar et al., 2020; Magobo et al., 2020; Mesini et al., 2020; Corzo-Leon et al., 2021; Fekkar et al., 2021; Thomaz et al., 2021; Díaz-García et al., 2022).

Overall, the global data indicate that *C. albicans* remains the predominant species identified in Candida infections. In this study, *C. albicans* was the most predominant species, nevertheless, NACS accounted for a high proportion (62.6%). Although reported resistance rates vary from study to study, the surveillance data collected suggest that azole resistance rates for *C. albicans* remain low (Pfaller et al., 2019; Nishimoto et al., 2020). However, *C. albicans* infections in the bloodstream pose a considerable threat in immunocompromised populations and the associated high mortality remains a major problem in the clinical setting. Therefore, *C. albicans* should not be overlooked as a serious public health threat. Remarkably, in this study, the percentage of FLC resistance in *C. albicans* was 13%, which is relatively high.

The acquisition of azole resistance is a serious concern given the limited number of molecules available for the treatment of IFIs. Moreover, this is much more worrying in resource-limited regions where FLC is the only available therapy (Kneale et al., 2016). As previously described, azole resistance is mainly conferred by mutations in the *ERG*11 gene and by the activity of efflux pumps. The *ERG*11 gene is highly polymorphic and more than 140 amino acid substitutions have been reported, indicating that this protein is very permissive to conformational changes. Most substitutions occur in three amino acid hotspot regions (105-165, 266-287 and 405-488), although mutations outside these regions can also be found (Debnath and Addya, 2014; Oliveira-Carvalho and Del Negro, 2014).

In the present study, 11 of the 19 FLC-resistant isolates harbored mutations which have been previously described in resistant isolates (T220L, Y132F, K143R) (Healey et al., 2018; Arastehfar et al., 2020). Notably, the T220L substitution was observed in one *C. albicans* isolate. In *C. auris*, five isolates carried the Y132F, and in the case of *C. parapsilosis* four isolates harbored the Y132F and one the K143R substitution.

The substitution of lysine for threonine at position 128 (K128T) was found in CAAL-A, CAAL-B and CAAL-D isolates, which is consistent with the findings of Cernicka et al., and Peron et al., who identified this amino acid substitution in FLC-resistant strains (Cernicka and Subik, 2006; Peron et al., 2016). However, several studies refute this association because K128T substitution has been found in multiple FLC-susceptible isolates, hence, in this study, we do not consider it as a mutation associated with resistance (Morio et al., 2010; Flowers et al., 2015). Nevertheless, it might influence translation efficiency, leading to alterations in protein production as it occurs with nearby mutations such as G129R and Y132F (Kumar et al., 2020).

According to Chow and coworkers, mutations contributing to FLC resistance are clade-specific in *C. auris*, being Y132F and K143R the most predominant in clade I, F126L in clade III, and Y132F in clade IV. However, Y132F in Erg11 is the most prevalent one (Chow et al., 2020). As described for isolates of clade IV (South America), we found only one mutation (i.e., Y132F). Concerning *C. parapsilosis*, high prevalence of the Y132F substitution was also noted in previous studies (Grossman et al., 2015; Choi et al., 2018). In addition, the latter suggested that isolates harbouring this mutation may have a higher propensity to cause clonal transmission and to persist in nosocomial settings (Thomaz et al., 2018). Regarding our isolates, the Y132F substitution was detected in four out of six *C. parapsilosis* isolates. However, considering the dates of collection of the isolates, It does not seem to be a clonal spread (**Table S3**). Although this report is due in 2023, some resistant isolates were obtained in 2015. Therefore, prior to the reports made in some parts of the globe (**Figure 5**).

Although previous reports indicate that the Mrr1 substitutions I283R, R479K, G583R, A854V, K873N and L986P are associated with FLC and/or VRC resistance, none of our isolates harbored them (Branco et al., 2015). Similar to Tac1, residues located near the C terminus (760, 761, 803, 956, and 966) might contribute to azole resistance (Nishimoto et al., 2020).

This study also illustrates that acquired azole resistance commonly relies on combined molecular mechanisms in clinical isolates (Morio et al., 2013). In addition to amino acid substitutions in Erg11, six of the 19 isolates also displayed active efflux activity. Interestingly, in two isolates lacking resistance-related mutations, efflux pumps activity was observed. Considering that efflux pumps play a key role in azole resistance, this might be the explanation for the observed phenotype. However, a further study evaluating the expression of all genes involved in efflux pump activity is required.

Regarding the isolates in which no mechanism for resistance was found, namely CAAL-A,B,D, CAAU-D, CAAU-D,E and CAPA-A, six novel Tac1 or Mrr1 substitutions (G52R, V366del, F682T, F683L, T695S and P229A) were identified in three of the six isolates. Moreover, four novel Erg11 substitutions (K22E, Q38T, F72V, Q77S) in the FLC resistant *C. albicans* (CAAL-B, CAAL-C) and *C. auris* (CAAU-B) were found. The putative role of these substitutions remains to be investigated. In isolates CAAL-A-D and CAAU-E, a different mechanism to those evaluated here should confer resistance; further analysis is ongoing.

Our study has some limitations. For instance, the antifungal susceptibility data obtained for antifungals other than FLC are not complete and were not performed by broth microdilution. The molecular basis of FLC resistance was investigated only by analysis of the *ERG*11, *TAC*1, and *MRR*1 genes, while other mechanisms conferring resistance, such as sterol composition and gene expression, were not investigated due to lack of funds. Finally, the clinical description of the patients was missing and will be reported alongside other patients infected by *Candida* antifungal resistant strains elsewhere.

Recently, the WHO released the fungal priority pathogens list (WHO FPPL) which includes *C. albicans, C auris* (Critical group), and *C. parapsilosis* (high group). The WHO describes that to overcome the lack of knowledge on infections caused by these fungi, more data and evidence on fungal infections and antifungal resistance to inform and improve response to FPP is needed (World Health Organization (WHO), 2022). Although our study does not have a large number of isolates and does not include other healthcare institutions, we provide evidence of Colombian isolates harboring resistance mutations to one of the most used molecules in the hospital setting.

In conclusion, we describe for the first time the presence of *Candida* spp isolates harboring Erg11 FLC resistant-related substitutions (Y132F, K143R and T220L) in patients admitted to a Colombian hospital. Although FLC-resistance rates differ significantly between countries and individual health facilities, the resistance rate in our study is relatively high, emphasizing the need for active surveillance to prevent further expansion of FLC-resistant *Candida* spp isolates in the clinical setting.

## Data availability statement

The datasets presented in this study can be found in online repositories. The names of the repository/repositories and accession number(s) can be found in the article/**Supplementary Material**.

## Author contributions

AP carried out the experiments. YV-C contributed to the development of efflux pumps protocol. AC-G, and AP analyzed the data. AC-G wrote the main manuscript. AP, SV-B, BA, and CP-G review and editing. and CP-G conceived the experiments and managed the resources. All authors contributed to the article and approved the submitted version.
