# Supplementary figures and images for: Emergence and circulation of azole-resistant C. albicans, C. auris and C. parapsilosis bloodstream isolates carrying Y132F, K143R or T220L Erg11p substitutions in Colombia
Source: Front Cell Infect Microbiol. 2023 Mar 21;13:1136217. doi: 10.3389/fcimb.2023.1136217 (PMC10070958; doi:10.3389/fcimb.2023.1136217)

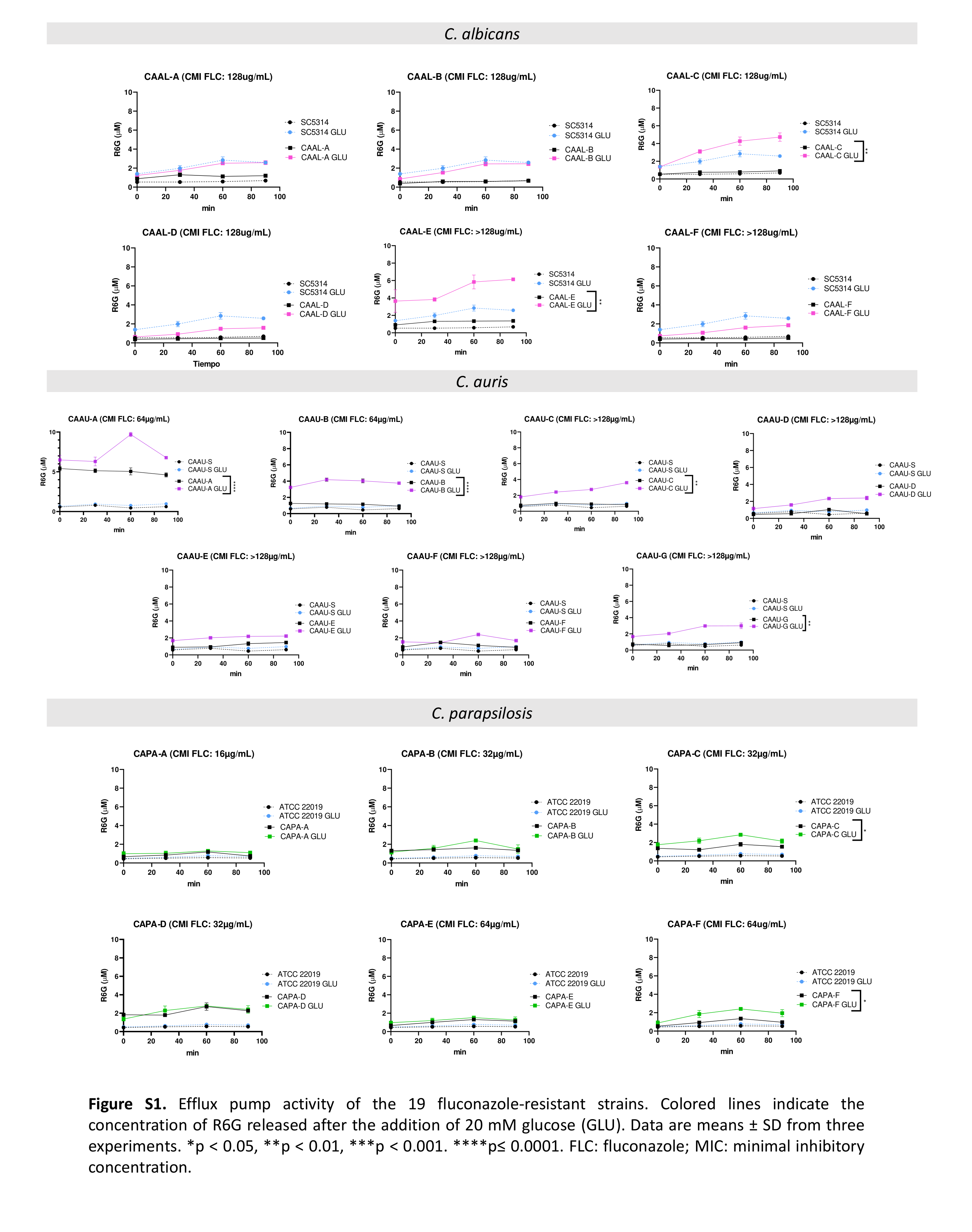

Supplement: Supplementary file 1 [file Image_1.tiff]
